# Genes and Genetic Pathways Regarding the Etiology and Pathogenesis of Ameloblastoma

**DOI:** 10.3390/genes17010065

**Published:** 2026-01-06

**Authors:** Vasileios Zisis, Petros Papadopoulos, Stylianos Papadopoulos, Konstantinos Poulopoulos, Christina Charisi, Dimitrios Parlitsis, Athanasios Poulopoulos

**Affiliations:** 1 Department of Oral Medicine and Pathology, School of Dentistry, Aristotle University of Thessaloniki, 54124 Thessaloniki, Greecestylianospapadopoul@gmail.com (S.P.); akpoul@dent.auth.gr (A.P.); 2 Department of Dentistry (Oral Medicine-Oral Pathology), School of Dentistry, European University Cyprus, Diogenous Street 6, Nicosia 2404, Cyprus

**Keywords:** genes, genetic, pathways, ameloblastoma, oral, tumor, odontogenic

## Abstract

**Background/Objectives:** Ameloblastoma is a benign odontogenic neoplasm characterized by locally aggressive behavior and frequent recurrences despite surgical treatment. It originates from odontogenic epithelium, including the cell rests of the dental lamina, remnants of the enamel organ, epithelial cell rests of Malassez, or the basal cell layer of the oral mucosa. Investigation of the etiopathogenesis of ameloblastoma has gained critical relevance due to the need for extensive surgical procedures, high recurrence rates, and its malignant potential. Accordingly, the aim of the present narrative review is to summarize current evidence regarding key aspects of ameloblastoma etiopathogenesis, with emphasis on signaling pathways, mutations, epigenetics, and epithelial–stromal interactions. **Methods:** An extensive literature search was conducted using the PubMed, Scopus, and Google Scholar databases, employing the keywords: “etiology”, “pathogenesis”, “molecular”, “biomarkers”, “cellular”, “epigenetic”, “mutation”, “pathway”, and “ameloblastoma”. In vitro studies, clinical studies, case reports, and narrative and systematic reviews published in English were included, without restriction on publication year. **Results:** Current evidence indicates that ameloblastoma pathogenesis is driven by dysregulation of multiple signaling pathways, particularly the MAPK and Sonic Hedgehog pathways, through recurrent activating *BRAF* and *SMO* mutations. In addition, alterations affecting the WNT/β-Catenin and PI3K/AKT signaling cascades, epigenetic modifications, and epithelial–stromal interactions, contribute to tumor behavior. **Conclusions:** Despite significant advances, genotype–phenotype correlations, mutation frequencies and coexistence, clonality, and other associations remain incompletely understood. Larger tumor cohorts and robust meta-analyses are required to clarify these associations and to leverage the development of personalized therapeutic strategies.

## 1. Introduction

Ameloblastoma is a benign, yet locally aggressive odontogenic neoplasm of the jaws, characterized by a high rate of recurrence despite surgical excision. It originates from the odontogenic epithelium, specifically from the cell rests of the dental lamina, the remnants of the enamel organ, the epithelial cell rests of Malassez, or the basal cell layer of the oral mucosa [1]. Additionally, cases of ameloblastoma, including, but not limited to the unicystic variant, arising from the epithelial lining of a cystic lesion, have also been reported [2,3]. Ameloblastoma typically presents at a mean age of 34 years and demonstrates a slight male predominance (53%). Although it is a relatively uncommon neoplasm, with a worldwide incidence of approximately 0.93 cases per million population [4], it accounts for nearly 10% of tumors affecting the jawbones. Moreover, depending on the population studied, ameloblastoma is considered either the most common, or the second most common odontogenic neoplasm, following odontomas or odontogenic keratocysts [5].

Odontogenic tumors constitute a highly diverse group of clinicopathological entities, ranging from completely benign lesions such as odontomas, to aggressive malignant neoplasms, and arising from the ectomesenchymal and/or epithelial tissues involved in normal tooth development [6]. Their classification represents a dynamic and evolving process that is frequently revised in light of emerging evidence [6,7]. According to the latest, 2022 World Health Organization (WHO) classification of Head and Neck Tumors, ameloblastoma is categorized into four distinct types, based on clinical and radiographic features: conventional (formerly referred to as solid or multicystic), unicystic, extraosseous/peripheral, and metastatic [7,8]. These variants account for approximately 75–85%, 13–21%, 1–4%, and less than 1% of cases, respectively [1]. This classification was already established in the previous 2017 edition, with the 2022 edition further including the entity of adenoid ameloblastoma among benign odontogenic neoplasms [6,7,8]. Adenoid ameloblastoma represents an extremely rare entity, with only a few dozen cases reported to date, and is histopathologically characterized by ameloblastoma-like epithelial components exhibiting cribriform architecture and duct-like structures, often accompanied by dentinoid formation [9]. Additionally, the clinicopathological entity of ameloblastic carcinoma is also recognized in the WHO classification [8]. Despite the similarity in nomenclature, ameloblastic carcinoma is distinct from metastatic ameloblastoma. It may arise de novo, or develop through malignant transformation of a pre-existing benign ameloblastoma [6]. Ameloblastic carcinoma is the most common malignant odontogenic neoplasm and is often characterized by aggressive clinical behavior and an unfavorable prognosis, partially attributable to its rarity, and the consequent lack of standardized treatment protocols [10]. In contrast, metastatic ameloblastoma is a histopathologically benign-appearing ameloblastoma that has nevertheless given rise to distant metastases [11].

Microscopically, ameloblastoma resembles the enamel organ of a developing tooth [12]. Conventional ameloblastoma is further subdivided into the follicular, plexiform, acanthomatous, granular, desmoplastic, and basal cell histological subtypes. Among these, the follicular and plexiform patterns are the most frequently encountered [1]. However, and particularly in light of more recent data regarding the clinical behavior of the desmoplastic subtype, it is now well-established that these histological variants do not exert a decisive influence on the biological, and consequently, the clinical behavior of the tumor [7]. Nevertheless, a large cohort study published in 2025 by Zheng et al. argues for the distinct classification of the desmoplastic type, in grounds of its particular genetic, radiological, anatomical, and prognostic characteristics [13].

The etiology and pathogenesis of ameloblastoma, as with neoplastic disease in general, are complex, and remain largely incompletely understood [1]. Nevertheless, numerous mutations, as well as genetic, epigenetic, and other molecular alterations, have been identified in neoplastic ameloblastoma cells. These alterations have the potential to disrupt the normal function of multiple signaling pathways and regulatory proteins, thereby promoting tumorigenesis, and accounting for the distinctively aggressive biological behavior of this neoplasm [12,14]. Disruption of the Mitogen-Activated Protein Kinase (MAPK) and Sonic Hedgehog (SHH) signaling pathways, most notably through activating mutations in the *BRAF* and *SMO* genes, which are discussed later, appears to play a central role in the pathogenesis and biological behavior of ameloblastoma [14].

In this context, although a wide range of immunohistochemical and molecular markers has been reported in ameloblastoma [15,16,17,18], only a limited subset appears to have prognostic or diagnostic value. Specifically, a recent review by Escobar-Duarte et al. identified the differential expression of 70 biomarkers, with the most notable among them being metalloproteinases, cytokines, and proteins associated with epithelial–mesenchymal transition (EMT), which serve as indicators of tumor invasiveness and aggressive clinical behavior [18]. In addition, the expression of calretinin, as well as the specific immunohistochemical patterns of cytokeratins, can aid in diagnosis, particularly in cases with ambiguous histology or limited tissue samples [19,20]. It should be noted that biomarker studies in ameloblastoma often exhibit heterogeneity in control tissues and are limited by small sample sizes, which can reduce reproducibility and comparability across studies [18]. More recently, in silico analyses of ameloblastoma biomarkers have emerged, leveraging computational and bioinformatics tools to analyze and visualize expression patterns as well as explore pathways and interactions by integrating multi-omics data, thus helping to identify novel diagnostic, prognostic, and therapeutic targets [21,22,23].

Furthermore, a recent systematic review by Kalogirou et al. [24] highlighted the involvement of cancer stem cells in the etiology and biological behavior of several odontogenic tumors, including ameloblastoma. At the same time, increasing attention has been directed toward the role of the interactions between neoplastic epithelial cells, and components of the extracellular matrix in tumor development and progression [25]. Finally, the possible contribution of oncogenic viruses, such as Epstein–Barr virus (EBV) and certain human papillomavirus (HPV) strains, to the etiology of ameloblastoma, remains controversial, although this association continues being investigated [26,27].

The study of the etiology and pathogenesis of ameloblastoma is not merely of theoretical interest. The need for extensive, often mutilating surgical procedures, the high recurrence rates despite surgical management, and the malignant or metastatic potential of ameloblastoma, have, in recent years, generated considerable interest in the potential use of pharmacological therapies for its treatment [12,14,16]. This shift is reflected in the WHO tumor classification system, which emphasizes the use of targeted therapies as complementary to traditional surgical management [8,28]. Although inhibition of various signaling pathways and regulatory proteins has been shown to suppress tumor growth in vitro, clinical experience in humans has thus far focused primarily on inhibitors of the Mitogen-Activated Protein Kinase (MAPK) pathway. These agents have been tested in patients with *BRAF* V600E-positive ameloblastoma, with encouraging results [16]. A recent review by Raemy et al. [29] documented the pharmacological treatment of 23 patients, 21 of whom harbored *BRAF* V600E-positive tumors. These patients were treated with MAPK pathway inhibitors, including dabrafenib, trametinib, vemurafenib, and cobimetinib, agents already widely used in the management of *BRAF* V600E-positive melanoma. A marked reduction in tumor size was observed in all patients, with four achieving complete radiological remission. In most reported cases to date, these treatments appear to be well-tolerated, with only mild adverse effects being described [29,30]. Other proposed pharmacological strategies include, among others, dual therapy with concurrent MAPK and Mouse Double Minute 2 (MDM2) inhibition [31], as well as targeting the Sonic Hedgehog and Phosphoinositide 3-kinase(PI3K)/AKT/Mammalian Target of Rapamycin (mTOR) signaling pathways [32,33,34]. Such approaches, may, on the one hand, help prevent the development of treatment resistant tumor cell clones, and on the other hand, enable the non-surgical treatment of patients with tumors of different genetic profiles.

In this context, the aim of the present narrative literature review is to present and synthesize current evidence on selected aspects of the etiology and pathogenesis of ameloblastoma, with particular emphasis on the major signaling pathways, genetic mutations, epigenetic modifications, and the interactions between neoplastic epithelial cells and the cells of the extracellular matrix.

## 2. Materials and Methods

### 2.1. Study Design and Literature Search Strategy

The present study was designed as a narrative, descriptive literature review, with the aim to synthesize and contextualize the most relevant, molecular, genetic, epigenetic, and microenvironmental evidence regarding the etiopathogenesis of ameloblastoma. A systematic review approach was not considered appropriate, given the marked heterogeneity of the available literature in terms of scope, methodology, and reported outcomes. Accordingly, the purpose of the present review is not to claim an exhaustive coverage of all existing knowledge, but to provide an integrated and reader-friendly overview of key mechanisms and emerging concepts regarding the etiology and pathogenesis of ameloblastoma.

A comprehensive literature search was conducted using the PubMed, Scopus, and Google Scholar databases, supplemented by a backward and forward citation tracking of relevant articles, in order to identify additional studies. The databases were accessed for the last time in November 2025.

The literature search was performed in two stages. In the first stage, broad search terms were used to obtain an overview of the existing ameloblastoma pathogenesis literature. These included general keywords such as “etiology”, “pathogenesis”, “molecular”, “genetic”, “mutation”, “epigenetic”, “polymorphism”, “pathway”, and “biomarkers”, combined with “ameloblastoma”.

In the second stage, more focused search queries were utilized to retrieve studies on specific molecular pathways, genes, and mechanisms. Search strings listed below are representative but not exhaustive, and were modified as needed across databases to capture relevant terminology and indexing differences:

1. (“sonic hedgehog” OR “SHH” OR “smoothened” OR “SMO” OR “patched” OR “PTCH” OR “GLI1” OR “GLI2” OR “GLI3”) AND “ameloblastoma”;
2. (“mitogen-activated protein kinase” OR “MAPK” OR “RAF” OR “BRAF” OR “RAS” OR “KRAS” OR “NRAS” OR “HRAS”) AND “ameloblastoma”;
3. (“WNT” OR “catenin” OR “CTNNB” OR “CTNNB1”) AND “ameloblastoma”;
4. (“phosphoinositide 3-kinase” OR “PI3K” OR “AKT” OR “mTOR” OR “PTEN”) AND “ameloblastoma”;
5. (“TP53” OR “p53” OR “MDM2”) AND “ameloblastoma”;
6. (“epigenetic” OR “methylation” OR “histone” OR “miRNA” OR “microRNA” OR “ncRNA” OR “lncRNA” OR “circRNA”) AND “ameloblastoma”;
7. (“microenvironment” OR “fibroblast” OR “myofibroblast” OR “alpha smooth muscle actin” OR “α-SMA”) AND “ameloblastoma”.

### 2.2. Inclusion Criteria and Study Selection

The present review included original research articles, in vitro, in vivo, and in silico studies, case reports, cohort studies, clinical trials, and previous descriptive and systematic reviews and meta-analyses published in English, and without restriction on publication year, in order to capture both landmark and recent contributions to the field.

Studies were considered eligible for inclusion if they provided relevant data on the molecular biology, genetic alterations, epigenetic modifications, or tumor–microenvironment interactions in ameloblastoma. Studies were included when they were considered representative, methodologically sound, or specifically influential for understanding of the field, with emphasis specifically placed on reports offering mechanistic insights, clinicomolecular correlations, or diagnostic and therapeutic implications. As the present review is a narrative review, aiming to descriptively analyze and synthesize heterogeneous evidence, no formal risk-of-bias (RoB) assessment was conducted.

### 2.3. Data Synthesis

Data synthesis was performed using a qualitative narrative approach. In this sense, selected studies were grouped according to their relevance to major biological themes, including signaling pathways, common genetic mutations, epigenetic mechanisms, and tumor–microenvironment interactions. The narrative synthesis was chosen to enhance conceptual clarity and accessibility for the reader while facilitating discussion of converging and diverging findings across studies.

## 3. Results and Discussion

### 3.1. Molecular Signaling Pathways in Ameloblastoma

The pathogenesis of ameloblastoma is increasingly recognized as being driven by the dysregulation of molecular signaling pathways that control fundamental cellular processes, such as proliferation, differentiation, survival and migration. These pathways, which are highly conserved across vertebrates, include the Mitogen-Activated Protein Kinase (MAPK), Sonic Hedgehog (SHH), WNT/β-Catenin, and Phosphoinositide 3-kinase (PI3K)/AKT pathways, among others. The following sections provide a detailed discussion on individual pathways, their alterations in ameloblastoma, and their potential contributions to tumor behavior and characteristics, while Table 1 summarizes the literature consensus and highlights areas of agreement.

### 3.2. Mitogen-Activated Protein Kinase (MAPK) Signaling Pathway

#### 3.2.1. Signaling Pathway Overview and Relevance to Ameloblastoma

Eukaryotic cells possess at least seven distinct mitogen-activated protein kinase (MAPK) signaling pathways, which are named after the terminal kinase of their respective signaling cascade. Despite their diversity, all MAPK pathways share a conserved core signaling architecture. In this cascade, a MAP kinase kinasekinase (MAP3K) phosphorylates and activates a MAP kinase kinase (MAP2K), which subsequently phosphorylates and activates a MAP kinase (MAPK) [35]. Within the MAPK/Extracellular Signal-Regulated Kinase (ERK) pathway, the most extensively studied MAPK signaling pathway in humans, a cell membrane receptor tyrosine kinase, such as the Epidermal Growth Factor Receptor (EGFR), is activated by mitogens, including growth factors, neurotransmitters, hormones, or other extracellular stimuli. Receptor activation leads to activation of RAS GTPases, which in turn activate serine/threonine kinases of the RAF family through GTP hydrolysis. Downstream, activated RAF, the MAP3K of this pathway, phosphorylates MEK1/2, which subsequently phosphorylates and activates ERK [14]. The latter two proteins then translocate to the nucleus, where they regulate the transcription of genes involved in cell proliferation, growth, metabolism, survival, migration and differentiation [35]. It is therefore evident that this pathway plays a central role in multiple physiological and pathological processes, including tumorigenesis, neurodegenerative diseases, and odontogenesis during embryonic development [55].

Between 2004 and 2013, a series of studies demonstrated [55] in vitro that ameloblastoma cell proliferation, growth, and survival, are promoted through activation of the MAPK/ERK pathway by molecules already known to be expressed at high concentrations in many cases of ameloblastoma. These include midkine, a heparin-bound growth factor that is normally expressed during odontogenesis, tumor necrosis factor-alpha (TNF-α), and the fibroblast growth factors FGF-7 and FGF-10.

#### 3.2.2. MAPK Pathway Mutations: The Role of BRAF V600E in Ameloblastoma Pathogenesis

In 2014, three independent research groups identified mutations in the *BRAF* gene in ameloblastoma cells in 46% (13/28) to 64% (54/84) of tumors examined, with the most common being the activating point mutation *BRAF* V600E [56]. These studies by Brown et al. [57], Sweeney et al. [58], and Kurppa et al. [59], have since been widely regarded as pivotal in advancing the understanding of the molecular pathogenesis of ameloblastoma [28]. *BRAF* V600E has been implicated in the pathogenesis of a wide spectrum of common and rare, solid and hematologic tumors [60]. It is detected in approximately 40–50% of melanomas and 10–70% of thyroid carcinomas, as well as in a subset of non-small cell lung cancers, colorectal cancers, and other malignancies [35,60], and has been associated with smoking and other environmental risk factors [61]. Functionally, *BRAF* V600E mutation results in constitutive activation of RAF kinase, thus rendering it independently of extracellular regulatory signals [14].

Sweeney et al. identified activating mutations in *SMO*, the gene encoding Smoothened protein of the Sonic Hedgehog pathway (discussed in detail in the next section), and *BRAF*, in 39% and 46% of 28 tumors studied, respectively [58]. According to the researchers, *BRAF* and *SMO* mutations are almost invariably mutually exclusive, giving rise to two distinct molecular subtypes of ameloblastoma, characterized by activating mutations in either the Sonic Hedgehog or MAPK signaling pathways. *BRAF* V600E-positive tumors were more frequently detected in the mandible in younger patients and were associated with a later recurrence compared with *SMO* mutated tumors, and with non-plexiform histological subtypes [56,58].

On the other hand, Brown et al. [57] detected activating *BRAF* mutations in 64% (54/84) of tumors studied, and argued that although *SMO* and *BRAF* mutation indeed rarely coexist, *SMO* mutations may occur rather secondarily to alterations in other components of the MAPK pathway. On this basis, they proposed that ameloblastoma does not comprise two distinct molecular subtypes, rather, disruption of the MAPK pathway constitutes the primary driving force in its etiology, with the proposed molecular “subtypes” of Sweeney et al. merely reflecting the presence or absence of activating *BRAF* mutations [56,57].

As will be discussed later, more recent studies seem to support the hypothesis of Brown et al., with the currently prevailing model suggesting that the *BRAF* V600E mutation, together with other, less frequent mutations affecting the MAPK pathway, represents one the most important, if not the principal, drivers of ameloblastoma pathogenesis [14,35,51]. However, this role should not be overstated, as MAPK pathway alterations alone are unlikely to fully account for the tumor’s etiology and its diverse clinical behavior [18].

The impact of *BRAF* V600E mutation on the demographic and clinicopathological characteristics of ameloblastoma and on its prognosis has received considerable attention from researchers conducting meta-analyses [36,37,38,39,40]. Specifically, a recent meta-analysis reviewing 833 tumors [36] identified the *BRAF* V600E mutation in 70.49% of ameloblastomas and demonstrated significant associations with mandibular location and younger patient age (under 54 years), but not with sex, histological subtype, or risk of recurrence. Furthermore, two different meta-analyses [37,38] of 937 and of 74 cases, respectively, similarly reported a higher prevalence of the *BRAF* V600E mutation in mandibular tumors and in younger patients while also identifying an association with unicysticameloblastomas. Finally, two separate focused meta-analyses did not associate *BRAF* V600E mutation with an increased risk of recurrence [39,40]. Interestingly, in a recently published cohort study by Zheng et al. [13], *BRAF* V600E mutations were detected in 93.5% (58/62) of desmoplastic ameloblastomas studied, with authors suggesting that the presence of this mutation contributes to defining desmoplastic ameloblastoma as a distinct subtype.

Nevertheless, it should be noted that the precise impact of *BRAF* V600E on the clinical behavior of ameloblastoma, particularly its aggressiveness, remains far from fully understood [35]. In vitro studies have suggested a more aggressive phenotype in *BRAF* V600E-positive tumors [62,63] while the underlying reasons for the preferential occurrence of this mutation in mandibular lesions have yet to be elucidated [56].

Regarding the biological consequences of *BRAF* V600E, Duarte-Andrade et al. [64] highlighted the fact that *BRAF* V600E-positive ameloblastomas exhibit a distinct metabolic profile from *BRAF* wild-type tumors, likely reflecting increased metabolic demands. Similar metabolic alterations have also been described in *BRAF* V600E-positive melanomas, colon carcinomas and thyroid carcinomas, and may be indicative of a more aggressive biological behavior.

Diagnostically, immunohistochemistry using the anti-BRAF V600E (VE1), which is specific for the mutant protein, demonstrates high sensitivity and specificity, comparable to molecular testing, making this method a readily-available and lower-cost option with implications for prognosis and treatment [65].

In addition to *BRAF* mutations, genetic alterations leading to constitutive activation of the MAPK/ERK pathway, such as activating mutations in Fibroblast Growth Factor Receptor 2 (*FGFR2*), or mutations in the *KRAS*, *NRAS* and *HRAS* genes, have been detected in approximately 2.5–5.5% of ameloblastomas [14]. These mutations frequently coexist with mutations in the *SMO* gene, but are almost always mutually exclusive with *BRAF* V600E. This pattern further underscores the previously noted pivotal role of a single activating mutation within the MAPK signaling pathway in the pathogenesis of a substantial proportion of ameloblastomas [35,56].

### 3.3. Sonic Hedgehog (SHH) Signaling Pathway

#### 3.3.1. Signaling Pathway Overview and Relevance to Ameloblastoma

The Sonic Hedgehog (SHH) signaling pathway is a highly conserved pathway among vertebrates and plays a crucial role during embryogenesis, where it regulates cell proliferation and differentiation through modulation of epithelium–mesenchymal interactions [66]. Consequently, it is involved in the organogenesis of most mammalian organs, including the teeth, and subsequently contributes to tissue regeneration and the maintenance of tissue homeostasis [67]. Given the close biological relationship between organogenesis and tumorigenesis, dysregulation of the SHH pathway has been documented in a wide range of neoplasms [32], including basal cell carcinoma of the skin, medulloblastoma, tumors of the pancreas, stomach, esophagus, prostate, as well as in inflammatory conditions [66].

In the absence of SHH, a glycoprotein with paracrine and autocrine activity, the transmembrane protein Patched-1 (PTCH1) represses Smoothened (SMO), a transmembrane G protein-coupled receptor [66]. Binding of SHH to PTCH1 inhibits this repression, resulting in phosphorylation and accumulation of SMO at the primary cilia of the cell membrane [67]. Subsequently, SMO catalyzes the dissociation of the suppressor protein Suppressor of Fused (SUFU) from the glioma-associated transcription factors GLI1, GLI2 and GLI3. These transcription factors then translocate to the nucleus, where they regulate the expression of genes involved in cell proliferation, survival, and differentiation [66].

In 2002, Heikinheimo et al. [68] reported the underexpression of SHH in ameloblastoma compared with the mesenchymal cells of the dental papilla. Subsequently, in 2004, Kumamoto et al. [69] observed higher expression of SHH pathway proteins in peripheral tumor cells compared with central tumor cells, suggesting the participation of these proteins in the pathogenesis of ameloblastoma through modulation of the epithelium–mesenchymal interactions. A series of later studies [11,70,71,72] confirmed activation of the SHH pathway in ameloblastoma and demonstrated differential expression of its component proteins within neoplastic cells. In particular, Filušová et al. [72] identified differences in the distribution and morphology of primary cilia among follicular, plexiform and basal cell histological subtypes in the 9 ameloblastomas they examined. A higher density of primary cilia was observed in follicular and plexiform ameloblastomas, whereas their presence was rare in the basal cell subtype.

#### 3.3.2. SHH Pathway Mutations: Smoothened (SMO) Mutations as a “Second Hit” in Ameloblastoma Pathogenesis

Mutations in the *SMO* gene, most commonly activating *SMO* L412F and *SMO* W535L, are detected in approximately 10% of ameloblastomas [14]. In their aforementioned 2014 study, Sweeny et al. [58] identified SMO mutations in 39% (11/28) of the tumors examined, while suggesting that tumors positive for *SMO* mutations, most commonly harboring the activating L412F mutation, also frequently detected in basal cell carcinoma of the skin, tend to occur in the maxilla, present at older ages, exhibit a higher recurrence rate, and preferentially display a plexiform histological pattern. Most of these observations are supported by a more recent study of 76 ameloblastomas by Gültekin et al. [41], which additionally reported a marked male predominance of 7:1 in patients with tumors harboring *SMO* mutations and also identified differences in the genetic profile of patients from different national backgrounds when comparing tumors from Europe and Turkey. Similarly, Awοtoye et al. [51] reported a higher frequency of *SMO* mutations in plexiform ameloblastomas and in tumors arising in the maxilla in their analysis of 10 ameloblastomas.

As already stated in the previous section, *SMO* mutations are almost always mutually exclusive with *BRAF* V600E mutation, while frequently coexisting with additional mutations, most commonly affecting the MAPK pathway, such as mutations in the Fibroblast Growth Factor Receptor-2 (*FGFR2*) gene, or the genes of the RAS family of proteins. Therefore, activating *SMO* mutations may represent a necessary “second hit”, required for neoplastic transformation and proliferation of ameloblastoma cells [41,42].

Additionally, *SMO* mutations appear to be rare, if present at all, in unicysticameloblastomas [41,73], while furthermore, You et al. [74] reported a lower frequency of *SMO* mutations in six cases of basal cell ameloblastomas compared to other histological subtypes. Recently, Zheng et al. [13] detected no activating SMO L412F mutations in the 62 cases of desmoplastic ameloblastomas they analyzed.

With regard to the *PTCH1* gene, Farias et al. [75] identified loss of heterozygosity (LOH) in ameloblastoma cells in 40% (4/10) of tumors examined, however, this alteration did not appear to affect the expression of the PTCH1, GLI1, or GLI2 proteins. Shimura et al. [43] reported a somatic *PTCH1* mutation in the neoplastic cells of a patient with invasive extraosseous/peripheral ameloblastoma, while Kawabata et al. [44] associated a *PTCH1* gene polymorphism, specifically a CGG trinucleotide repeat in the 5’ untranslated region, with an increased risk of developing ameloblastoma in their case–control study of 28 ameloblastoma patients and 70 healthy controls. Finally, the suppressor mutation *PTCH1* K729M was identified [45] in a case of unicystic ameloblastoma arising in a patient with Gorlin syndrome. It should be noted that *PTCH1* mutations have also been documented in odontogenic keratocysts, both sporadic, and Gorlin syndrome-associated [32].

### 3.4. WNT/β-Catenin Signaling Pathway

#### 3.4.1. Pathway Overview and Biological Relevance

Signaling through the WNT/β-catenin pathway plays a pivotal role at all stages of embryogenesis in a wide range of organisms, including during tooth development [35], and also contributes to tissue regeneration and homeostasis. Dysregulation of this pathway is implicated in disease, as numerous somatic mutations affecting its component genes contribute to the development of various solid tumors and hematopoietic malignancies [76].

In the absence of WNT ligands, a family of 19 small, extracellular proteins with autocrine or paracrine activity, β-catenin is sequestered by the cytoplasmic “destruction complex”. This complex consists of the proteins: Adenomatous Polyposis Coli (APC), Axin, Casein Kinase 1 (CK1), and Glycogen Synthase Kinase 3 (GSK3), which, combined, promote the proteasomal degradation of β-catenin. Upon binding of WNT to the Frizzled (FZD) receptor and the Low-Density Lipoprotein Receptor-Related Proteins 5 and 6 (LRP5/6) of the cell membrane, the destruction complex is recruited to the membrane, thereby releasing β-catenin, which can accumulate in the cytoplasm. The latter will subsequently translocate to the nucleus, where it interacts with transcription factors to activate the genes involved in cell proliferation, differentiation, survival, maintenance of clonality, and modulation of inflammatory responses [76,77,78].

In addition, the WNT/β-catenin pathway contributes to the regulation of the MAPK signaling cascade [35]. When the destruction complex is active, GSK3 phosphorylates and inactivates the RAS proteins, thereby exerting an inhibitory effect on MAPK signaling.

#### 3.4.2. WNT/β-Catenin Signaling in Ameloblastoma

In ameloblastoma, differential expression of several WNT family proteins, such as: WNT1, WNT2, WNT5a, WNT8a, WNT8b, and WNT10a, has been demonstrated in comparison with other tissues [11,79,80,81,82].

Chatterjee et al. [46], in their systematic review including 586 ameloblastoma cases, noted that β-catenin localization was predominantly membranous and cytoplasmic, rather than nuclear, within the epithelial neoplastic cells. However, cases exhibiting increased β-catenin nuclear expression were more frequently associated with conventional and metastatic ameloblastomas, as well as with follicular, desmoplastic, and acanthomatous histological subtypes and appeared to correlate with tumor growth, invasion, metastatic potential, and an increased risk of recurrence [46,47,48].

Liu et al. [83] demonstrated in vitro, that miR-29a-3p, a microRNA overexpressed in ameloblastoma, exerts a suppressive effect on the β-catenin-interacting protein gene (*CTNNBIP1*), a negative regulator of β-catenin signaling. Similarly, Zhang et al. [84] reported that interleukin-8 (IL-8), also overexpressed by epithelial and certain stromal cells of conventional ameloblastoma, can induce activation of β-catenin expression. Yang et al. [85] furthermore showed that increased hydrostatic pressure, a common feature in ameloblastomas, activates the WNT/β-catenin pathway, leading to overproduction of, among others factors, matrix metalloproteinases 2 and 9 (MMP-2 and MMP-9), thereby promoting angiogenesis, tumor growth, and of course, the infiltration of adjacent tissues. On this basis, the authors proposed marsupialization as a preoperative therapeutic approach aimed at reducing intracystic pressure prior to definitive surgical tumor resection.

In ameloblastoma, activating mutations in the β-catenin gene (*CTNNB1*) are considered generally infrequent, and in approximately half of the reported cases, they coexist with the *BRAF* V600E mutation [14]. Additionally, Ning Li et al. and Siriwardena et al., have also reported [49,50] *APC* tumor suppressor mutations, which are also observed in approximately 85% of sporadic colorectal cancer cases, with allele frequencies of 6.25% to 27.5% in 30 patients, and in 50% (3/6) of patients, respectively. Finally, mutations affecting the gene encoding the LRP6 receptor have also been reported to coexist with *BRAF* V600E mutation [14].

### 3.5. PI3K/AKT Signaling Pathway

#### 3.5.1. Pathway Overview and Biological Function

The Phosphoinositide 3-kinase (PI3K)/AKT signaling pathway is a highly conserved intracellular signaling network in eukaryotic cells that promotes cell survival, growth, and cell cycle progression [86]. This pathway is functionally complementary to the MAPK pathway, and activation of one pathway typically results in negative regulatory feedback on the other [87].

Among the various PI3K isoforms present in eukaryotic cells, class IA PI3Ks have been most strongly and consistently implicated in cancer pathogenesis [86,87]. These kinases are heterodimers, composed of a catalytic and a regulatory subunit, and may be activated by several upstream signals, including tyrosine kinase receptors, such as those involved in MAPK signaling, insulin receptors, and G protein-coupled receptors [88]. Activated class IA PI3Ks catalyze the conversion of Phosphatidylinositol-4,5-biphosphate (PIP2) membrane phospholipids to Phosphatidylinositol-3,4,5-triphosphate (PIP3) by adding a phosphate group at the 3′ position of their inositol ring. This lipid modification creates the docking sites necessary for the binding of the PI3K-dependent kinases PDK1 and PDK2, which subsequently phosphorylate and activate AKT, a serine/threonine kinase [88]. At this stage, AKT activation may be antagonized by the tumor suppressor protein Phosphatase and Tensin Homolog (PTEN) [89]. Phosphorylated AKT has many substrates and therefore can exert pleiotropic effects, including inhibition of programmed cell death through inactivation of BCL-2-associated X protein (BAX), suppression of the tumor suppressor protein Forkhead box O (FOXO), and activation of the Mammalian Target of Rapamycin (mTOR), which enhances anabolic metabolism and protein synthesis of the cell by acting on the ribosomes [87,88].

#### 3.5.2. PI3K/AKT Signaling in Ameloblastoma

As previously discussed in the context of MAPK pathway signaling, studies conducted between 2004 and 2005 by Sandra et al. and Hendarmin et al. [90,91] demonstrated the ability of midkine and TNF-α to activate the PI3K/AKT pathway through induction of AKT phosphorylation, thus promoting proliferation and survival of ameloblastoma cells in vitro.

In 2004, Nodit et al. [92] reported the loss of at least one allele encoding PTEN in 62% of the neoplastic cells of the 15 ameloblastomas and ameloblastic carcinomas examined, with such losses occurring more frequently in mandibular neoplasms. In addition, Kumamoto et al. in 2007, ref. [93] demonstrated the expression of phosphorylated AKT, PI3K, and PTEN in the epithelial neoplastic cells of 40 ameloblastomas, and observed increased PI3K/AKT pathway activity in the plexiform, compared with the follicular histological subtype, as well as in neoplastic epithelial cells, compared with normal oral mucosa. Conversely, PTEN activity was reduced in tumor cells.

More recently, Ning Li et al., analyzed 80 cases of ameloblastoma, and observed increased AKT expression in the tissue samples exhibiting malignant transformation [52]. The same group further associated phosphorylated mTOR expression with tumor invasiveness and a higher risk of recurrence in a study of 85 ameloblastomas [53]. Chaisuparat et al., in an analysis of 30 cases, detected phosphorylated AKT at higher frequencies in ameloblastomas than in odontogenic cysts and odontogenic keratocysts, however, they did not identify significant correlations with the anatomical localization, or histological subtype [54].

Finally, mutations in *PTEN*, and *PIK3CA*, the gene encoding the catalytic subunit of PI3K, have been detected in ameloblastomas, often coexisting with mutations in *BRAF* or *SMO* [14,51]. Narayan et al. identified mutations in exon 5 of *PTEN* in 25% (5/20) of ameloblastomas they examined [94]. Three of these were silent mutations, one constituted a point mutation that resulted in a single nucleotide substitution, and the last one resulted in a premature stop codon. Additionally, Lapthanasupkul et al. reported methylation of 65% (13/20) of examined *PTEN* gene promoters, however, this epigenetic modification was not statistically associated with changes in gene expression [95].

### 3.6. Other Selected Mutations and Their Potential Effects

Recent studies have identified additional mutations beyond those already discussed, many of which tend to coexist with the *BRAF* V600E mutation [14,56]. Several of these mutations occur in genes that have previously been implicated in various types of malignancy, however, available studies have not always been able to determine whether, and to what extent, these genetic alterations influence the biology, and consequently the clinical behavior of ameloblastoma [57,61,96,97]. According to the recent review by Marín-Márquez et al., somatic mutations detected in ameloblastoma cells include alterations in the following genes: *EGFR*, *KMT2D, ROS1*, *HSPA4*, *TP53*, *ANKRD31*, *CDC73*, *DHX29*, *SCN5A*, *SMARCB1*, *CREBBP*, *PLEKHN1*, *GNAS*, *CLTC*, *NES*, and *BCOR* [14].

Below, data regarding the most extensively studied of these mutations and their potential biological effects are presented:

#### 3.6.1. TP53

The *TP53* gene encodes p53, a key tumor suppressor protein, whose deregulation has been associated with a wide spectrum of malignancies [15]. Several studies have reported reduced or absent p53 expression in ameloblastoma, however, mutations in *TP53* itself are detected only infrequently [98], and their overall impact is considered to be of limited importance [17]. Instead, suppression of p53 activity appears to be mediated primarily through the overexpression of MDM2, a negative regulator of p53, a phenomenon that is commonly observed in ameloblastoma [99,100,101,102]. Two studies [103,104] identified *TP53* mutations in 2 out of 29, and 1 out of 20 cases, respectively, whereas Migaldi et al. [105] failed to detect any *TP53* microsatellite mutations in the genomes of 24 ameloblastomas examined. Interestingly, *TP53* mutations have been more consistently reported in ameloblastic carcinomas [104,106], with at least one case report suggesting that a *TP53* mutation might have contributed to the malignant transformation and metastatic potential of the tumor [107].

#### 3.6.2. KMTD2

Mutations in the Histone-lysine N-methyltransferase-2D (*KMT2D*) gene have been reported by five studies [51,57,61,96,97], including frameshift changes, premature stop codons, and small nucleotide insertions and deletions, with reported frequencies ranging from 20% (2/10) to 36% (5/14) of all analyzed tumors. Notably, Awotoye et al. identified *KMTD2* mutations in 2 out of 4 follicular ameloblastomas, but in none of the 6 plexiform tumors examined [51]. Histone-lysine methyltransferase-2D is a transcriptional coactivator with oncogenic potential [108]. Mutations in *KMT2D* are frequently detected across abroad range of malignancies, yet data regarding the impact of the overexpression of the encoded protein on tumor progression remain inconsistent and controversial [108].

#### 3.6.3. CDC73

Mutations in the Cell Division Cycle-73 (*CDC73*) gene, which encodes parafibromin, a protein involved in cell cycle regulation, and whose dysregulation is often observed in parathyroid carcinomas and in ossifying fibromas associated with the hyperparathyroidism-jaw tumor syndrome (HPT-JT), were recurrently identified in 2 out of 10 ameloblastomas studied by Guan et al. [61]. These mutations were characterized by a low variant allele frequency (VAF), leading the authors to suggest that *CDC73* mutations may be confined to specific subclonal populations of ameloblastoma cells.

#### 3.6.4. HSPA4

Recurrent mutations in the Heat Shock Protein-4 (*HSPA4*) gene were detected in 2 out of 4 neoplasms examined by Shi et al. [96]. The protein encoded by this gene is known to suppress programmed cell death and to enhance tumor aggressiveness in several cancer types. It should be noted, that the overexpression of other proteins of the heat shock protein family has also been consistently documented in ameloblastoma [17].

### 3.7. Epigenetic Modifications

Epigenetic modifications comprise a group of mechanisms through which gene expression is regulated, without alterations in the DNA sequence itself, as occurs with mutations. Instead, these mechanisms involve changes in the structure and the configuration of chromatin, that is, DNA and histones, as well as post-transcriptional regulation, primarily at the mRNA level [109]. Unlike mutations, epigenetic modifications are reversible. Although they occur physiologically in organisms during normal development, their disruption is also associated with the pathogenesis of various neoplastic diseases [110].

The principal epigenetic modification mechanisms include: DNA methylation, histone modifications, and non-coding RNAs (ncRNAs). DNA methylation is mediated by DNA methyltransferases (DNMTs), a group of proteins, which add a methyl group to the cytosines within CpG dinucleotides. These CpG sites are abundant in the promoter region of approximately 60% of human genes, and subsequently, their methylation typically leads to transcriptional suppression [109]. Histone modifications involve, on the one hand, post-translational changes, such as acetylation, methylation, phosphorylation, that can either activate, or silence the genes surrounded by the histones, and, on the other hand, alterations in the nucleosome structure [111]. Finally, ncRNAs regulate gene expression by influencing mRNA splicing and stability, DNA methylation, nucleosome modification, and genomic imprinting [112].

A systematic review by Santos et al., including studies published up to 2019, identified 11 studies demonstrating gene hypermethylation or hypomethylation associated with tumorigenesis or tumor aggressiveness in ameloblastoma, as well as differential expression of DNMTs and various types of ncRNAs, with potential roles in the etiology and pathogenesis of the tumor [110]. Among the most significant findings were the hypomethylation of the matrix metalloproteinase 9 (*MMP9*) gene, and the hypermethylation of the genes encoding the tumor suppressors p16 and p21.

Table 2 summarizes the most recent in vitro studies (published after 2019) with regard to epigenetic modifications in ameloblastoma, however, epigenetic alterations affecting specific signaling pathways have already been discussed in their respective sections.

Additionally, Abiko et al. reported an interesting case of ameloblastoma undergoing malignant transformation into ameloblastic carcinoma, in which methylation of the tumor suppressor gene encoding p16 was detected exclusively in the tumor regions exhibiting malignant histopathological features. The authors suggested that this epigenetic modification may be responsible for the malignant transformation [126].

### 3.8. Cells of the Extracellular Matrix

The abundant extracellular matrix of ameloblastoma contains fibroblasts, myofibroblasts, osteoblasts, immune cells, and other stromal components. These cells interact with the neoplastic epithelial cells [25] by inducing the secretion of growth factors, interleukins, osteolytic cytokines, and extracellular matrix-degrading enzymes [25,127]. Collectively, these factors contribute to tumor growth, and to the migratory and invasive capabilities of ameloblastoma. Similar epithelial–stromal interactions have been described in other neoplasms [25], nevertheless, it is not always clear whether these mediators are predominantly produced by the epithelial or the stromal components of the tumor [128].

In 2014, Fuchigami et al. [129] demonstrated that interleukin-1α (IL-1α), expressed by epithelial ameloblastoma cells, stimulates stromal fibroblasts to secrete interleukin-6 and 8 (IL-6 and IL-8), which in turn promote neoplastic cell proliferation and motility. More recently, Yoshimoto et al. [128] observed that bidirectional interactions between neoplastic epithelial cells and fibroblasts induce IL-6 secretion by both cell types, leading to increased expression of the osteoclast differentiation factor Receptor Activator of Nuclear factor Kappa B Ligand (RANKL) by the epithelial tumor cells. Both Fuchigami et al. and Bakkalci et al. [130,131] demonstrated in vitro that fibroblasts enhance the invasive capacity of ameloblastoma cells. The latter group further showed [132] that the composition of the extracellular matrix decisively influences the transcriptional profile of ameloblastoma cells. Specifically, a fibroblast-rich matrix promotes the overexpression of genes associated with extracellular matrix degradation, while an osteoblast-rich matrix induces the overexpression of certain oncogenes and the repression of tumor suppressor genes. Intercellular signaling between osteoblasts and ameloblastic parenchyma has likewise been shown to increase tumor invasiveness, proliferation, and migratory capacity [133].

The presence of myofibroblasts within the tumor stroma and their secretion of cytokines and growth factors has been linked to increased invasiveness in multiple neoplasms [25]. However, data regarding the density of alpha-smooth muscle actin (α-SMA)-positive myofibroblasts in ameloblastoma, compared with other odontogenic tumors or cysts, as well as among different ameloblastoma subtypes, remain conflicting [134,135,136,137,138,139,140]. Notably, Fregnari et al. [141] associated increased stromal α-SMA expression with MMP-2 overexpression and cortical bone infiltration, suggesting a more aggressive tumor phenotype. Additionally, the localization of α-SMA expression within the central epithelial islands, rather than exclusively in the extracellular matrix, has been identified as a histopathological feature distinguishing ameloblastoma from ameloblastic carcinoma [142,143,144].

### 3.9. Limitations and Future Directions

Despite significant progress in shedding light on the molecular landscape of ameloblastoma during the last decade, several limitations inherent to the current body of evidence should be acknowledged for reasons of methodological transparency.

First, the present review was intentionally designed as a narrative, rather than a systematic review. This decision reflects, on the one hand, the marked heterogeneity of the available literature, both in scope and methodology, which spans, among other aspects, signaling pathways, genetic and epigenetic alterations, and interactions within the tumor microenvironment. Rather than pursuing an exhaustive quantitative synthesis, emphasis was placed on integrating high-quality, recent and earlier, representative and landmark studies, in an accessible and conceptually cohesive manner, aiming to provide the reader with a clear bird’s eye view on the current landscape of ameloblastoma research.

A persistent challenge identified across individual studies, and which can only partly be addressed by meta-analyses, relates to the relatively small sample sizes, and the often limited representation of histological subtypes and other patient clinical and demographic characteristics. These limitations definitively constrain the strength and reliability of clinicomolecular associations reported in the literature. Future research would indeed benefit from larger, prospective, multi-center cohorts with standardized clinical data collection, long-term follow-up, and uniform molecular profiling.

Finally, even though targeted therapy in *BRAF* V600E-positive ameloblastoma has generated considerable interest and encouraging clinical results, as already discussed, alterations of the MAPK pathway alone are unlikely to fully explain the biological behavior and clinical variability of this neoplasm. Accumulating evidence implicating additional signaling pathways, epigenetic mechanisms, and tumor–microenvironment interactions underscores the need for prospective clinical trials exploring non-MAPK pharmacological targets, as well as combination therapeutic approaches, in order to advance towards personalized and precise treatment strategies for combating ameloblastoma.

## 4. Conclusions

Ameloblastoma is the most common, or the second most common, odontogenic neoplasm of the jaws. It is characterized by benign histology but exhibits locally invasive behavior and frequent recurrences despite surgical treatment. The etiology and pathogenesis of ameloblastoma are multifactorial, and its clinical behavior is directly influenced by its molecular biology. In this context, the dysregulation of signaling pathways, alterations in tumor suppressor and oncogenic proteins, and uncontrolled expression of enzymes that promote extracellular matrix metabolism and osteoclastogenesis, can explain its invasive nature. Mutations in the MAPK pathway, particularly the activating *BRAF* V600E mutation, and mutations in the *SMO* gene of the Sonic Hedgehog signaling pathway, are considered pivotal in the pathogenesis of ameloblastoma. Disruption of other signaling pathways and genes, epigenetic modifications, and interactions between epithelial and stromal cells, also contribute to the tumor’s occurrence and behavior. However, genotype–phenotype correlations, mutation frequencies and coexistence, clonality, and other associations, remain uncertain or relatively unknown. In this context, studies with larger tumor cohorts as well as statistical meta-analyses of existing data are required. Current research on the pharmacological treatment of ameloblastoma primarily focuses on treating mostly *BRAF* V600E-positive tumors with MAPK pathway inhibitors. These treatments are generally well-tolerated, however, these findings are primarily derived from case reports and small series, and further studies are required to confirm their safety and efficacy. Additionally, testing drugs with different molecular targets is necessary, both to prevent the development of resistance in certain cell clones and to treat patients whose tumors have a different genetic profile.

## Figures and Tables

**Table 1:** Summary of key molecular signaling pathways implicated in ameloblastoma.

Molecular Pathway	Key Mutations/Frequency	Clinicopathological Associations	References
**MAPK**	Activating *BRAF* V600E mutation in ~70% of ameloblastomas. Activating *FGFR2*, *KRAS*, *NRAS*, and *HRAS* mutations in ~2.5–5.5%.	Considered the principal molecular driver of ameloblastoma. *BRAF* V600E-postive tumors are consistently associated with mandibular location and younger patient age. Possible association with unicysticameloblastomas. No association with sex, histological subtype, or recurrence risk. Impact on tumor aggressiveness remains incompletely defined.	[35,36,37,38,39,40]
**Sonic Hedgehog (SHH)**	Activating *SMO* mutations in ~10% of ameloblastomas. *PTCH1* silencing mutations and polymorphisms reported.	*SMO* activating mutations very rarely coexist with *BRAF* V600E, but often occur in background of other MAPK mutations, possibly acting as a “second-hit”. More frequently reported in maxillary tumors, however, meta-analyses and larger cohorts are needed for consistent genotype–phenotype associations.	[14,41,42,43,44,45]
**WNT/β-Catenin**	Activating *CTNNB1* mutations are uncommon and often coexist with *BRAF* V600E. *APC* silencing mutations reported.	May be implicated in a subset of ameloblastomas. Cytoplasmic β-catenin expression observed in most tumors. Nuclear β-catenin expression, present in a minority of cases, has been associated with conventional and metastatic ameloblastomas, aggressive behavior, and increased recurrence risk. Meta-analyses and larger cohorts are needed for consistent genotype–phenotype associations.	[14,46,47,48,49,50]
**PI3K/AKT**	*PTEN* and *PIK3CA* mutations are uncommon and frequently coexist with *BRAF* or *SMO* mutations.	May be implicated in a subset of ameloblastomas. Increased PI3K/AKT/mTOR pathway activity has been associated with aggressive tumor behavior, malignant transformation, and recurrence risk. Meta-analyses and larger cohorts are needed for consistent genotype–phenotype associations.	[14,51,52,53,54]

**Table 2:** Recent (published after 2019) studies regarding epigenetic modifications in ameloblastoma.

Reference	Number and Type of Neoplastic and Control Tissue Samples	Results
Carvalho et al. (2025) [113]	30 ameloblastoma, 15 odontogenic keratocyst, 10 adenomatoid odontogenic tumor, 8 odontogenic fibroma, 8 calcifying odontogenic cyst, 10 odontogenic myxoma, and 6 ameloblastic fibroma samples	Acetylation of histones H2A on lysine 5 (H2AK5ac) and H3 on lysine 27 (H3K27ac) is reduced in aggressive odontogenic lesions compared with non-aggressive lesions. Ameloblastoma exhibited lower expression levels of these markers relative to less aggressive tumors.
Chen et al. (2020) [114]	6 ameloblastoma tissue samples and 6 healthy oral tissue samples	Underexpression of miRNA miR-524-5p results in overexpression of interleukin-33 (IL-33) and its receptor, ST2, by both epithelial neoplastic cells and stromal lymphocytes. These proteins contribute to the immune response of the tumor and microenvironment modulation.
do Amaral-Silva et al. (2021) [115]	38 ameloblastoma samples, 6 ameloblastic carcinoma samples, and 10 dental follicle samples	DNMT3B was overexpressed in ameloblastomas and ameloblastic carcinomas compared with dental follicles. DNMT1, DNMT3A, and H3K9ac were overexpressed in ameloblastic carcinomas compared with ameloblastomas. DNMT1 overexpression was associated with specific clinical parameters and the *BRAF* V600E mutation, while DNMT3B overexpression was associated with an increased risk of tumor recurrence.
do Amaral-Silva et al. (2022) [116]	10 ameloblastoma tissue samples and 10 dental follicle samples	No differences were observed in the methylation status of genes encoding MutS family DNA repair proteins. However, methylation was strongly associated with reduced expression of the MSH2 and MSH6 proteins in ameloblastoma.
Guan et al. (2020) [117]	96 ameloblastoma tissue samples and 15 healthy oral tissue samples	miRNA miR-141-3p is significantly underexpressed in ameloblastoma, leading to overexpression of NCAM1, a protein associated with cell adhesion and increased tumor invasiveness.
Liu et al. (2022) [118]	Not specified	6 types of circular RNA (circRNA) were identified. circRNA circ_0089153 acts as a competing endogenous RNA for miRNA miR-608, which suppresses EGFR and p53 expression.
Niu et al. (2020) [119]	3 ameloblastoma tissue samples and 3 healthy oral tissue samples	Detection of 3.673 differentially methylated adenosine-m6A sites in mRNAs (16.2% hypermethylated), 4.975 in lncRNAs (29.4% hypermethylated), and 364 in circRNAs (22.5% hypermethylated).
Niu et al. (2021) [120]	104 ameloblastoma tissue samples and 20 healthy oral tissue samples	miRNA miR-1-3p, which limits cell proliferation, migration, and tumor invasive capacity, is underexpressed in ameloblastoma.
Phattarataratip and Lam-Ubol (2025) [121]	30 ameloblastoma, 30 odontogenic keratocyst, 30 adenomatoid odontogenic tumor, and 15 dental follicle samples	Odontogenic cysts and tumors exhibit reduced levels of histone H3 trimethylation at lysine residue 9 (H3K9me3) compared with dental follicles, with ameloblastoma showing the second lowest H3K9me3 levels among these lesions. H3K9me3 levels in ameloblastomas demonstrate marked heterogeneity among cases and its upregulation may be linked to ameloblastoma multilocularity.
Pongpanich et al. (2021) [122]	5 ameloblastoma tissue samples and 3 dental follicle samples	A total of 25.255 differentially methylated CpG dinucleotides and 17 differentially methylated CpG islands were identified. Six CpG islands were associated with the genes *BAIAP2*, *DUSP6*, *FGFR2*, *FOXF2*, *NID2*, and *PAK6*.
Sun et al. (2020) [123]	6 ameloblastoma tissue samples and 6 healthy oral tissue samples	lncRNA ENST00000512916 is overexpressed in ameloblastoma. Its suppression inhibits cell proliferation and migration, as well as the expression of CDK2, CDK4, and CDK6 kinases.
Udompatanakorn et al. (2025) [124]	30 ameloblastoma, 20 odontogenic keratocyst, 20 dentigerous cyst, and 6 dental follicle samples	Higher expression of α-ketoglutarate-dependent dioxygenase homolog 5 (ALKBH5), a N6-methyladenosine (m6A) eraser, was observed in ameloblastomas and odontogenic keratocysts compared with dental follicles and dentigerous cysts.
Wang et al. (2024) [125]	76 ameloblastoma tissue samples and 30 healthy oral tissue samples	Modification of tRNA guanine to N-7 methylguanosine by METTL1 activates the MAPK pathway and is associated with a higher risk of recurrence.

## Data Availability

No new data were created or analyzed in this study. Data sharing is not applicable to this article.

## Abbreviations

α-SMA	Alpha smooth muscle actin
AKT	Protein kinase B (ΡΚΒ)
ALKBH5	AlkB homolog 5, RNA demethylase
ANKRD31	Ankyrin repeat domain 31
APC	Adenomatous polyposis coli
BAIAP2	Brain-specific angiogenesis inhibitor 1-associated protein 2
BAX	BCL2-associated X protein
BCOR	BCL6 corepressor
BRAF	v-raf murine sarcoma viral oncogene homolog B
CDC73	Cell division cycle 73 (Parafibromin)
CDK2	Cyclin-dependent kinase 2
CDK4	Cyclin-dependent kinase 4
CDK6	Cyclin-dependent kinase 6
circRNA circ_0089153	Circular RNA circ_0089153
CK1	Casein kinase 1
CLTC	Clathrin heavy chain
CpG	5’-cytosine-phosphate-guanine-3’
CREBBP	CREB-binding protein
CTNNB1	Catenin beta 1
CTNNBIP1	Catenin beta-interacting protein 1
DHX29	DExH-box helicase 29
DNMT1	DNA methyltransferase 1
DNMT3A	DNA methyltransferase 3 alpha
DNMT3B	DNA methyltransferase 3 beta
DUSP6	Dual specificity phosphatase 6
EGFR	Epidermal growth factor receptor
ERK	Extracellular signal-regulated kinase
FGF-7	Fibroblast Growth Factor 7
FGF-10	Fibroblast Growth Factor 10
FGFR2	Fibroblast growth factor receptor 2
FOXF2	Forkhead box F2
FOXO	Forkhead box O transcription factor
FZD	Frizzled
GLI	Glioma associated
GLI1	GLI family zinc finger 1
GLI2	GLI family zinc finger 2
GLI3	GLI family zinc finger 3
GNAS	Guanine Nucleotide-binding protein, Alpha stimulating
GSK3	Glycogen synthase kinase 3
H2AK5ac	Histone 2A lysine 5 acetylation
H3K9ac	Histone H3 lysine 9 acetylation
H3K9me3	Histone H3 lysine 9 trimethylation
H3K27ac	Histone H3 lysine 27 acetylation
HRAS	Harvey rat sarcoma virus
HSPA4	Heat shock protein family A member 4
IL-1α	Interleukin 1 alpha
IL-6	Interleukin 6
IL-8	Interleukin 8
IL-33	Interleukin 33
KMT2D	Histone-lysine N-methyltransferase-2D
KRAS	Kristen rat sarcoma
lncRNA	Long non-coding RNA
lncRNA ENST00000512916	Long non-coding RNA ENST00000512916
LRP5	Low-density lipoprotein receptor-related protein 5
LRP6	Low-density lipoprotein receptor-related protein 6
MAP2K	Mitogen-Activated Protein Kinase Kinase
MAP3K	Mitogen-Activated Protein Kinase KinaseKinase
MAPK	Mitogen-Activated Protein Kinase
MDM2	Mouse double minute 2 homolog
MEK1/2	MAPK/ERK Kinase 1/2 (MAP2K)
METTL1	Methyltransferase-like 1
miR-1-3p	MicroRNA 1-3p
miR-29a-3p	MicroRNA 29a-3p
miR-141-3p	MicroRNA 141-3p
miR-524-5p	MicroRNA 524-5p
MMP-2	Matrix metalloproteinase 2
MMP-9	Matrix metalloproteinase 9
mTOR	Mammalian target of rapamycin
MSH2	MutS homolog 2
MSH6	MutS homolog 6
NCAM1	Neural Cell Adhesion Molecule 1 (CD56)
ncRNA	Non-coding RNA
NES	Nestin
NID2	Nidogen 2
NRAS	Neuroblastoma RAS viral oncogene homolog
PAK6	p21-activated kinase 6
PDK1	PI3K-dependent kinase 1
PDK2	PI3K-dependent kinase 2
PI3K	Phosphoinositide 3-kinase
PIK3CA	Phosphatidylinositol-4,5-bisphosphate 3-kinase catalytic subunit alpha
PIP2	Phosphatidylinositol-4,5-biphosphate
PIP3	Phosphatidylinositol-3,4,5-triphosphate
PLEKHN1	Pleckstrin homology domain containing N1
PTCH1	Patched 1
PTEN	Phosphatase and tensin homolog
p16	Cyclin-dependent kinase inhibitor 2A **(CDKN2A)**
p21	Cyclin-dependent kinase inhibitor 1A **(CDKN1A)**
p53	Tumor protein p53
RAF	Rapidly Accelerated Fibrosarcoma
RANKL	Receptor activator of nuclear factor kappa B ligand
RAS	Rat sarcoma virus
RAS GTPase	RAS Guanosine Triphosphatase
ROS1	ROS proto-oncogene 1, receptor tyrosine kinase
SCN5A	Sodium voltage-gated channel alpha subunit 5
SHH	Sonic hedgehog
SMARCB1	SWI/SNF-related matrix-associated actin-dependent regulator of chromatin subfamily B member 1
SMO	Smoothened
ST2	Suppressor of Tumorigenicity 2
SUFU	Suppressor of fused
TNF-α	Tumor Necrosis Factor-alpha
TP53	Tumor protein p53 gene
VE1	*BRAF* V600E mutation-specific antigen
WNT	Wingless-related integration site protein
